# Supplementary material for: Coherent noise enables probabilistic sequence replay in spiking neuronal networks
Source: PLoS Comput Biol. 2023 May 2;19(5):e1010989. doi: 10.1371/journal.pcbi.1010989 (PMC10153753; doi:10.1371/journal.pcbi.1010989)
Supplement: Attachment — Submitted filename: reply_letter.pdf [file pcbi.1010989.s007.pdf]

Younes Bouhadjar
Institute of Neuroscience and
Medicine (INM-6) &
Peter Grünberg Institut (PGI-7,10)
Jülich Research Centre
D-52425 Jülich
Germany

phone: +491778728402
email: y.bouhadjar@fz-juelich.de

Boris S. Gutkin and Samuel Gershman
Editorial Board Member, PLoS Computational Biology

Jülich
January 30, 2023

Resubmission of manuscript PCOMPBIOL-D-21-02013

Dear Boris S. Gutkin and Samuel Gershman,

please find enclosed our revised manuscript "*Coherent noise enables probabilistic sequence replay in spiking neuronal networks*", which we resubmit for publication in PLoS Computational Biology. All concerns of the reviewers have been addressed, and the manuscript was changed accordingly. A point-by-point reply for each reviewer is provided below on separate pages.

As requested, we resubmit two versions of the revised manuscript with and without change tracking markup.

Best regards,

Younes Bouhadjar (on behalf of all authors)

encl
- reply to reviewer #1
- reply to reviewer #2
- revised manuscript with highlighted changes
- revised manuscript without change highlighting

Reply to comments of reviewer #1

[R1:1] *. . . Overall, the paper is well written, easy to follow, and well supported by experiments and figures.*

We thank the reviewer for the thorough read and the constructive comments.

[R1:2] *The main message is rather simplistic and could probably be expressed much more concisely without the detailed mechanisms of the example network that is being used in this manuscript. The authors even note themselves that "The problem of noise averaging and the proposed solution are not unique to the model presented here" so it could be made a bit clearer why this complex model needs to be introduced in order to make the points presented. Generally, I think the paper could be shortened in a few places but overall, it shows the effect of correlated noise in a nicely structured manner on an interesting example.*

The central questions put forward in this study are

- how a biological neuronal network can perform sequential memory recall in the presence of ambiguous stimuli,
- "how the statistics of learned sequences affect decision making", and
- "how decision strategies can be adjusted after learning" in a natural manner.

We cannot address these questions without a model that can learn and process complex, partly overlapping sequences. The spiking TM model introduced in (Bouhadjar et al., 2022) is one of the few existing, biologically plausible spiking neuronal network models that can do this. The problem of noise averaging is a secondary problem we faced and solved during our work on this. It is an important aspect, but not the central theme. We agree that a manuscript specifically dedicated to the "noise averaging" problem could have been written in a more generic setting. But this is not our intention.

We agree, however, that the original manuscript was not as concise as it could have been. In the revised manuscript, we shortened the text in several places. See also our replies to the comments below and the revised manuscript containing change tracking mark up.

[R1:3] *The example setup on which you demonstrate these results is nice and simplistic, which is good. However, I am still a bit curious whether you tested this in a bit more complex scenario where the network learns more than 2-3 sequences which can overlap and will share transition weights in more places than the first two elements. For instance, what if there are a lot of sequences that contain an F to C transition?*

For illustration, we restricted this study to relatively simple sets of sequences. In (Bouhadjar et al., 2022), we have shown that the spiking TM model can learn more complex sequence sets with more and longer sequences, and larger overlaps. A systematic study of the network capacity will be subject of future work. In the revised manuscript, we added a small paragraph on this at the end of the Discussion. In general, probability matching becomes harder for a larger number of sequences for the reasons explained in this new paragraph. However, we have added a new supplementary figure demonstrating approximate probability matching for an additional exemplary set of 5 competing sequences.

[R1:4] *Line 200 with "feeds" do you mean activates?*

The term "Feeds" in this context refers to the external inputs being provided to the neuron. The neurons that receive this type of input may not be activated due to the inhibition. We changed slightly the text to improve clarity.

[R1:5] *Sentences after like 200 are difficult to follow if you are not deep into the topic. They contain a lot of loaded terms. Is it possible to rewrite this a bit simpler or refer to some references here? I know you mention the materials and methods section but if this is needed to understand this paragraph, I would probably just remove it here.*

To make the text more comprehensible, we shortened the text and added references to the equations describing the neuron dynamics.

[R1:6] *Figure 2: Does it matter in which order the sequences are presented during training?*

We included in the manuscript at the end of section "Methods and Materials" in the paragraph "External inputs during learning" a description of the order in which we present the sequences during training. For a different, for example, randomized, order, the plasticity dynamics are different but the overall behavior remains the same (see new supplementary figure S5 in the revised manuscript).

[R1:7] *Why do you present F? Would this produce the same results if you present sequences ABD and ACE?*

We chose this setting specifically to highlight decision-making during sequence replay, where the decision is made long after the cue, during which the network activity is sparse. In our study, the decision is made during the transition from "F" to "B" or from "F" to "C". After learning, the response to "F" is sparse, however, the response to "A" is non-sparse because it is the first character in the sequence. The same thing would work without "F", provided the learning rates are adjusted to account for the fact the network response to "A" is non-sparse (see figure below).

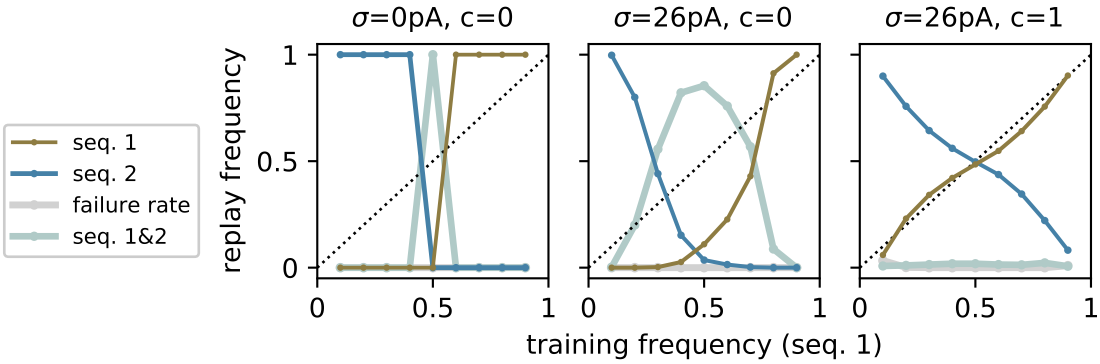

Replay statistics for sequences {A,C,E} and {A,B,D}. Dependence of the relative replay frequencies of sequences 1 (brown) and 2 (blue), the failure rate (gray), and the joint probability of replaying both sequences (silver) on the training frequency of sequence 1. Left, middle, and right panels depict results for three different noise configurations $\sigma = 0\,\text{pA}$, $c = 0$ (left), $\sigma = 26\,\text{pA}$, $c = 0$ (middle), and $\sigma = 26\,\text{pA}$, $c = 1$ (right). Circles represent the mean across $N_\text{t} = 151$ trials, averaged across 5 different network realizations. Learning rates: $\lambda_+ = 0.00018$, $\lambda_- = 0.0000028$, and $\lambda_\text{h} = 0.00016$.

[R1:8] *Line 243: How many neurons in a subpopulation fire usually? 50% seems like a low threshold. Is it common for many neurons in the subpopulation not to fire? How many neurons can fire in other populations if for instance D is recognized? It would be interesting to have a bit more details or statistics to get an intuition here.*

We thank the reviewer for this question, because it led to the discovery of a mistake in the description: we define a sequence to be successfully replayed if more than $0.5\rho = 10$ in the subpopulation corresponding to the last element in that sequence fire, rather than 50% of all neurons in that subpopulation (which would be 75 for a subpopulation size of 150). Here, $\rho = 20$ is the minimal number of neurons that is required to trigger the WTA circuit. We have fixed this in the paragraph above equation (1) and in the 2nd paragraph of the first subsection in Results. Note that this mistake did not affect the results, only the description in the text. In addition, we added a paragraph to "Task performance measures" in the Methods defining successful replay.

The parameter ρ defines the minimal number of active neurons in a subpopulation after successful learning. In (Bouhadjar et al., 2022), we have shown that the actual number of active neurons in a subpopulation after successful learning is indeed close to ρ in the absence of noise (provided the learning rate is not too high). We find a similar behavior in the presence of correlated noise, as shown in the new supplementary figure S2.

[R1:9] *Figure 3: does this show actual results or is this a hypothetical visualization? You present stimulus A only 3 times, are the outcomes picked to be representative for the general distribution? In the correlated noise example, I would assume it could also happen that the distribution on 3 examples is not 1:2.*

The spiking activity shown in Fig.3 are actual simulation results. Panels C–D show the spike responses in three consecutive trials (cue presentations). We show only three trials in each panel to make sure the details of the spike response (such as the spike dispersion in panel C) remain visible. For the cases shown in C and D, there is variability in the responses across trials and network realizations. In Fig.3, the starting trials were handpicked to demonstrate the typical (representative) behavior within three trials. For the case shown in Fig.3C, the decisions are approximately 1:2 on average. The full picture, i.e., the statistics for a large number of trials and network realizations, is provided in Fig.4. In the revised manuscript, we added a note on this in the caption of Fig.3.

[R1:10] *Figure 3 C: Can you elaborate more on why there are fewer red dots (inhibitory activations) in the second row?*

This is due to the failure of the WTA which is explained in the following text from the manuscript at the beginning of the section "Noise canceling cannot be overcome by increasing noise amplitude:

"In the presence of uncorrelated noise with high amplitude, the spikes from all neurons, in all competing subpopulations, are similarly dispersed. A large dispersion in spike times prohibits a fast and reliable activation of inhibition by one of the competing subpopulations."

[R1:11] *Figure 3 A: Do all arrows to one subpopulation have the same weight wCF?*

In Fig.3A, each arrow represents a larger number of connections between all neurons in the pre- and postsynaptic populations. The thickness of the arrows represents the average synaptic weight across all these connections. In the revised manuscript, we left a note on this in the main text

and in the caption of Fig.3.

On average, the connections from F to C are stronger than those from F to B, as the combination {FC} is shown more often during learning (see main text).

For a given pair of populations, e.g., F and C, the average weights are not exactly identical as

a) the synaptic weights are randomly initialized (before learning; see "Initial conditions and network realizations" in Methods and Table 1), and
b) each neuron in C receives a slightly different number of inputs from B due to the random and sparse connectivity (see Results, 2nd paragraph, and Methods:Network Model:Network structure).

This variability is however negligible, and therefore not shown in Fig.3.

[R1:12] *Figure 3 caption: I don't think you can assume people know what WTA means.*

Thank you for bringing this to our attention. We introduced the winner-take-all (WTA) mechanisms explicitly in the first section of the results and introduced the abbreviation again in Figure 3.

[R1:13] *Lines 247-253 seem a bit repetitive to what has already been said earlier*

We agree and have shortened this paragraph in the revised manuscript.

Note that we have moved the definition of the response latency t^x to this paragraph, in response to comment R2:17 of reviewer #2.

[R1:14] *Line 255: What determines this if there is no noise?*

Data is shown across (different trials as well as) different network realizations, i.e, different connectivities. For some network realizations, the WTA is not fast enough even for $p = 0.4$ to suppress the activity of the less frequent sequence. Thus both sequences are replayed simultaneously.

[R1:15] *Line 263 missing word "that" at end of line*

Thank you, we have corrected this now.

[R1:16] *Line 267: You don't have noise during learning, do you have a reason why this is a plausible assumption to make? Do you have an idea what effect correlated noise during learning would have?*

We think that the assumption of vanishing noise during training is a) not entirely implausible and b) not critical. In the Discussion of the revised manuscript, we explain this in a new paragraph addressing this subject.

[R1:17] *2 paragraphs starting from line 270 seem to repeat a lot of information from the beginning (but adding formulas) maybe it can be cut down in places*

We have revised and slightly shortened this text, and hope that all redundancy is gone now. The paragraph around equation (2) is the first mentioning of the noise averaging problem in the

Results section. Similarly, the following paragraph demonstrates for the first time in the Results section that correlations can easily resolve the problem.

[R1:18] *Figure 4 caption: What distinguishes the "5 different network realizations"?*

The network realizations differ in their (sparse) connectivity structure and in the initial synaptic weights. Both the connectivity and the initial synaptic weights are randomly and independently generated (see "Network realizations and initial conditions" in "Methods: Network model", and "Initial conditions and network realizations" in Table 1).

[R1:19] *Chapter starting in line 319: The paragraphs jump between biology and your results a bit. After reading the start of the first paragraph I was wondering "can you assume control over noise? Where would this come from?" You allude to that towards the end of the second paragraph. Maybe you could combine the biology. Results after line 329 seem to be a bit repetitive again, maybe that could be cut a bit shorter.*

We fully agree with the reviewer and have revised and shortened this and the first part of the following section. We hope that the line of reasoning and the link to biology is clearer now.

[R1:20] *Line 333: What to you mean with "changing the effective weights"?*

By "changing the effective weights", we mean changing the neuronal excitability. For example, this could be achieved by means of neurotransmitters or spatiotemporal oscillations. We agree that the use of the term "effective weight" was ambiguous, and replaced it by "excitability".

[R1:21] *Line 345-345: Do you have a source for that? How does your mechanism work if they become locked after some observations?*

Cortical waves can occur both spontaneously or locked to internally generated events such as saccades during active vision. In the latter case, the external cue (e.g., the visual input at the onset of the fixation after a saccade) is locked to the saccade-triggered oscillation. This systematic locking of the stimulus to the oscillation phase would lead to a systematic, non-exploratory replay of only one of the competing sequences (not necessarily the one with the highest training frequency). In the case of spontaneous waves, however, the external stimulus is not systematically locked to the oscillation. In this study, we are exploiting this second form of waves to generate across-trial variability, and hence, exploratory behavior. We have added a more detailed discussion (including references) on this in the 3rd paragraph of the Discussion and revised the 1st paragraph in "Random stimulus locking to spatiotemporal oscillations…".

[R1:22] *Lines 348-350: This is the same setup you explained already, right?*

Yes, it is the same setup. We made this clearer now in the manuscript and further shortened the text.

[R1:23] *Lines 363-364: I'm not sure I understand this, can you maybe elaborate a bit more what you mean here?*

The phrasing was somewhat misleading here. We corrected this in the revised manuscript and hope it is clearer now. What we are saying in this sentence is that the model's replay strategy

can be changed by adjusting the oscillation amplitude for a range of physiological frequencies from alpha to gamma.

[R1:24] *Lines 365-368: A bit confusing to me. It seems like a clear conclusion is missing here.*

We agree that the original text was somewhat misleading. We have revised this paragraph and added a conclusion.

Reply to comments of reviewer #2

[R2:1] *... The present study provides highly valuable insight and makes a contribution at both, the conceptual and mechanistic level, showing how switching defined noise characteristics in a simple spiking network model allows to control switching between decision strategies. This study very well fits the scope of PLOS CB and deserves publication in revised form. Below I provide a number of major review comments to be addressed during revision that are mainly concerned with the description of methods that lack clarity and transparency in several parts, and with the biological realism of the model (including single neuron biophysics) that currently is neither transparently described nor discussed.*

We thank the reviewer for the thorough reading, the positive evaluation, and the constructive comments. Please find our responses to these comments below.

[R2:2] **1. Additional questions to the model** *Here I formulate a few questions that could possibly be addressed in an extended model analysis to provide more insight in model function and capacity; these are suggested additions and I do not expect or require the authors to address all of them.*

We thank the reviewer for giving us the opportunity to address some of the many remaining questions. We have performed additional simulations to investigate points (I), and (III) below. The remaining points (II), (IV) and (V) have, at least to some extent, been addressed in (Bouhadjar et al., 2022). Details are given below.

[R2:3] *(I) What is the capacity in terms of number of sequences to be represented in probability matching?*

For illustration, we restricted this study to relatively simple sets of sequences. In (Bouhadjar et al., 2022), we have shown that the spiking TM model can learn more complex sequence sets with more and longer sequences, and larger overlaps. A systematic study of the network capacity will be subject of future work. In the revised manuscript, we added a small paragraph on this at the end of the Discussion. In general, probability becomes harder for a larger number of sequences for the reasons explained in this new paragraph. However, we have added a new supplementary figure demonstrating approximate probability matching for an additional exemplary set of 5 competing sequences.

[R2:4] *How does performance depend on the number of training runs?*

We investigated the learning progress in terms of the prediction error (time-to-solution) in (Bouhadjar et al., 2022) for different sequence sets. In terms of probability matching, the learning progress depends on the parameters of the task. For the sequence set I used here with $p = 0.2$, and noise parameters $\sigma = 20$ pA and $c = 1$, for example, the replay frequencies match the training frequency after approximately 20 training episodes (see figure below).

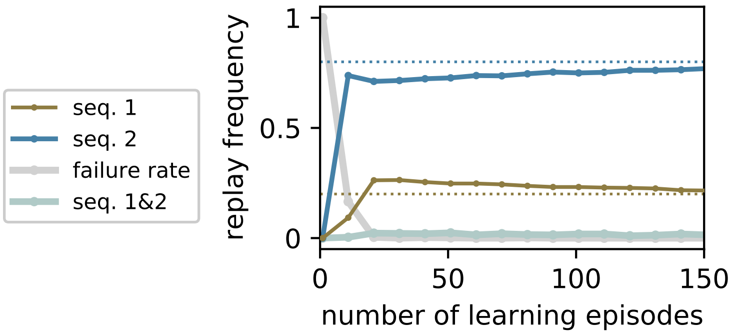

Influence of the number of training episodes on the replay performance. Dependence of the replay frequencies of sequences 1 (brown) and 2 (blue), the failure rate (gray) and the joint probability of replaying both sequences (silver) on the number of training episodes. An "episode" refers to a set of ten sequences, where each sequence is picked from the set $\{s_1, s_2\}$ with relative frequencies $p_1 = 0.2$ (brown dotted horizontal line) and $p_2 = 1 - p_1 = 0.8$ (blue dotted horizontal line), respectively.

[R2:5] *(II) Currently the sequence lengths is four cues from a set of six (corresponding to six populations). What is the capacity of the model in terms of sequence length (more than 4) and performance for sequences where the same cues reoccur, e.g. AABB?*

With respect to the capacity in terms of sequence length and overlap, see our response to R2:3. Sequence with recurring characters immediately following each other (such as in ABBC) cannot be learned with the current version of the model. In the revised manuscript, we have added a new paragraph at the end of the Discussion where this is explained in detail. We are currently working on a modification of the model to permit the learning of such sequences.

[R2:6] *Does a larger number of populations (e.g. 12 instead of six) alter any of the conclusions (i.e. can this model be scaled to a larger set of syllables).*

In the version of the spiking TM model used in this study, each character is assigned to one subpopulation, i.e., the number of subpopulations is identical to the size of the alphabet (for sequence set I, we need 6 characters, so the number of subpopulations is 6; for a sequence set II in Fig.5, the number of subpopulations is 8). However, the model is scalable in the sense that the number of subpopulations per character can be altered. The original TM model of Hawkins & Ahmad (2016), for example, assigns 40 subpopulations to a single character, each comprising 32 neurons. This larger amount of neuronal resources leads to a larger number of possible patterns that can be stored in the network, as discussed in detail in (Ahmad & Hawkins, 2016). The sequence set used in (Hawkins & Ahmad, 2016) is however of comparable (or even somewhat lower) complexity as the one used in (Bouhadjar et al., 2022). We have shown in (Bouhadjar et al., 2022) that the original and the spiking TM model perform similarly well in terms of both the sequence prediction error and the time-to-solution. In other words, for the benchmarking sequence set used in (Hawkins & Ahmad, 2016), one subpopulation per character is sufficient.

[R2:7] *(III) How 'badly' does the model perform or how much would the results deviate if the STDP rule was not disabled during replay? If it would still perform reasonable, then this would be an interesting result and conceptually better aligned with theories of continual learning.*

We tested this by performing additional simulations and presenting the results in a new supple-

mentary figure S3. With intact plasticity during replay, the probability matching performance gradually degrades. This is primarily a consequence of the fast autonomous sequence replay. In the revised manuscript, we explain this in detail in a new paragraph in the Discussion.

[R2:8] *(IV) What are the necessary conditions under which the network can be trained successfully? Can the interstimulus interval within a sequence easily be changed (e.g. to 25ms instead of 50ms) without loss of function? Are any of the neuron / synapse / STDP parameters matched to the 50ms inter-stimulus interval within a sequence? I think this is an important point that should be addressed as generally we would expect sequential task to vary strongly in inter-stimulus duration while network and synapse parameters can be expected to be fixed.*

We thank the reviewer for bringing this up. In (Bouhadjar et al., 2022), we have shown that the spiking TM model is robust with respect to changes in the sequence speed. Even for fixed synaptic and neuronal parameters (including STDP parameters), the model can successfully learn sequences for a range of inter-stimulus intervals. In the revised manuscript, we added a sentence on this feature at the beginning of the introduction.

For realistic parameters, the range of tolerable inter-stimulus intervals spans values between about 10 and 100 ms. The lower bound is mainly determined by spike transmission delays, synaptic and membrane time constants. The upper bound is primarily given by the duration of the plateau potential caused by NMDA spikes (dAPs), but also by the time constant of the STDP potentiation. As discussed in (Bouhadjar et al., 2022), longer inter-stimulus intervals would require an additional working memory mechanism. We are currently investigating this in a follow-up study.

[R2:9] *(V) l.267: "During training, the weak noise employed here hardly affects the network behavior as the external inputs (stimulus) are strong and lead to a reliable, immediate responses." If the noise level was not altered but kept constant throughout learning and replay, what are the conditions of successful learning with respect to the stimulus? Currently the stimulus is deterministic and reliable enforcing a single spike in each stimulated neuron, this seems a rather unrealistic condition in the brain.*

We think that the assumption of vanishing noise during training is a) not entirely implausible and b) not critical. In the Discussion of the revised manuscript, we explain this in a new paragraph addressing this subject. In (Bouhadjar et al., 2022), we demonstrated that the spiking TM model can successfully learn sequences in the presence of moderate levels of background noise and non-synchronous external inputs with randomly jittered spike times.

[R2:10] 2. Learning protocol and task *The definition of the learning protocol in l.99ff is poor. How often are sequences presented overall during training, this is neither mentioned here not in the main text? Is this depending on the relative frequencies of the sequences or/and on the number of different sequences (e.g. 2 vs. 3 different sequences) or is always the same total number of training trials applied?*

The reviewer is right that this was unclear. We improved the text in the section "Materials and methods" subsection "Learning protocol and task" to address the open questions.

[R2:11] *What is the performance and strategy for few trial learning (in the order of 10)?*

As shown in (Bouhadjar et al., 2022), the sequence prediction performance depends on the

complexity of the sequences to be learned. For two sequences and the model parametrization used in this study, the performance errors drop to zero after about 25 presentations (Fig. 8 in Bouhadjar et al., 2022). For 6 sequences, it takes about 40 presentations (Fig. 9 in Bouhadjar et al., 2022). In the figure shown above in our response to comment R2:4, we assess the probability matching performance for two competing sequences. In this example, the probability matching performance has converged after about $N_\mathrm{e} = 20$ training episodes with $p_1 = 0.2$ and $p_2 = 0.8$ (20 episodes correspond to $N_\mathrm{e} \cdot L \cdot p_1 = 40$ presentations of the sequence 1 and $N_\mathrm{e} \cdot L \cdot p_2 = 160$ presentations of the sequence 2, where $L = 10$ is the number of presented sequences in each training episode). The replay frequency of sequence 2 matches the training frequency already after about $N_\mathrm{e} = 10$ training episodes, corresponding to $N_\mathrm{e} \cdot L \cdot p_2 = 80$ presentation, i.e., sequence 2 converges sooner because it is shown more frequently.

[R2:12] *The description of the task in l.99ff and of the task performance in l.177ff is also unclear. I understand that there are three strategies that can be desirable and thus as low or high performance would be dependent on the strategy. The four basic measures in l.181 are thus rather counting statistics of sequences that subserve derived quantification of task performance?*

We agree that the measures defined in the subsection on "task performance measures" under section "Materials and methods" are rather describing the replay statistics. We have changed the title of this subsection accordingly to "Sequence replay statistics". The task is mainly described in Fig.2 and the main text in subsection "A spiking neural network recalls sequences in response to ambiguous cues:

"We refer to the "maximum probability" strategy (Fig. 2B, left) as the case where the network exclusively replays the sequence with the highest occurrence frequency during training. When adopting the "probability matching" strategy, the network replays sequences with a frequency that matches the training frequency (Fig. 2B, middle). The "full exploration" strategy refers to the case where all sequences are randomly replayed with the same frequency, irrespective of the training frequency (Fig. 2B, right)"

We further added a note on this in the task description in the section "Learning protocol and task" under the section "Materials and methods". For the conclusions of this work, it is sufficient to show the replay frequencies defined in the Methods subsection "Sequence replay statistics". One could define specific replay performance measures for each replay strategy, but this is not necessary for this study.

[R2:13] 3. Changes from the learning to the replay network *The Results text states: "The model can be configured into a replay mode, where the network autonomously replays learned sequences in response to a cue stimulus. This is achieved by changing the excitability of the neurons such that the activation of a dAP alone can cause the neurons to fire" (l.227f). My understanding of the re-configuration from training to replay mode is a considerably more drastic one and this must be made more explicit and transparent! When switching from learning to replay 'mode' the authors device at least FOUR major changes to the network model. (I) The STDP is disabled (what is a biologically plausible mechanism, dependency of STDP on neuromodulatory input? Is there any experimental evidence? How does replay perform if STDP is not disabled?); (II) external noise is now providing background while there was zero noise during learning; (III) The inhibitory synaptic weight is decreased by almost a factor of 6 (now still about 4 times larger than the excitatory weight); (IV) Excitatory weights are reduced by a factor of about 2. How do these changes translate in the statement above on the activation by dAP alone? Did I miss any*

other modifications? This switch of operational 'mode' and change in basic parameters is a very strong supervised modification of the network and the biophysics of the synapses. This needs to be described transparently in the Method section and it must at least be briefly described in the Result section, possibly also in the caption of Fig. 1 or by means of an additional sketch in Fig. 1. Please discuss the biological plausibility / potential mechanisms for each change that underlies the switch of the network 'mode'.

We followed the reviewer's advice and made the difference between the learning and the replay mode more transparent in the revised manuscript.

Specifically, we mention more explicitly that plasticity is switched off during replay (point I) in the 1st subsection of the Results (paragraph "The model can be configured into replay mode ..."), and in the caption of Fig.1C. In the revised Discussion, we added a new paragraph (paragraph "The spiking TM model employed in this study...") explaining why we removed plasticity during replay. Briefly: due to the high replay speed, the potentiation of connections between neurons representing consecutive sequence elements would become too strong, and the weights would be driven into saturation. With the exponential time dependence used in the current model, the short inter-element intervals during replay lead to a substantially larger potentiation as compared to the potentiation during learning where the time intervals are longer. One potential solution would be to alter the STDP timing dependence such that pre-post spike-time intervals that are comparable to the inter-element intervals during replay (or shorter) lead to a weaker potentiation. See also our reply to to R2:7 above.

Further, we made clearer that background noise is injected during replay but not during learning (point II) in the 1st subsection of the Results (paragraph "To foster exploratory behavior..."), in the Methods (paragraph "External inputs during replay..."), and in the caption of Fig. 1C. In a new paragraph in the Discussion (paragraph "In this study, we equip the network with noise..."), we explain the underlying rationale, and point to (Bouhadjar et al., 2022) where we have investigated the sequence learning performance in the presence of noise. See also our reply to R1:16 and R2:9.

The points III (change in inhibitory weight) and IV (change excitatory weights) are not important, except point III for the supplementary figure S1. In the original version of our manuscript, we reduced the strength of inhibition and excitation to account for the increased excitability (reduced thresholds) of excitatory neurons during the replay. But we totally agree with the reviewer that these additional changes require an explanation of the potential underlying biophysical mechanisms. A reduction in the inhibitory and excitatory weights during replay leads to a larger range of response latencies (Fig. 4), and thereby to a more gradual representation of the training frequencies. The same effect can however also be achieved by increasing the time constant τ_{EE} of the alpha-function shaped excitatory synaptic currents. In the revised manuscript, we increased τ_{EE} from $17\,\mathrm{ms}$ to $30\,\mathrm{ms}$ (both during learning and replay). With this adjustment, the modifications III and IV become not necessary for high correlation levels. In the revised manuscript, we therefore repeated all simulations without III and IV, and updated the respective figures in the main text accordingly. For S1 Fig, we adjust only the inhibitory synaptic weight during replay from the inhibitory neuron to the subpopulation F. The purpose here was to show that the model can perform, in principle, probability matching for intermediate levels of noise correlation if certain parameters are adjusted, but we are not aware of a particular biological mechanism that can explain this. We added a note on that in the subsection "Noise amplitude and level of correlation control replay strategy". We argue anyway in the manuscript that high and intermediate levels of correlation cannot be explained by the type of noise discussed in this subsection and suggested

an alternative type of noise.

[R2:14] 4. Role and plausibility of inhibition. *l.160 "each inhibitory spike prevents all excitatory neurons in the network that have not generated a spike yet from firing. " - the description as „not ... yet " is unclear; is spiking artificially prevented for the rest of the input sequence/trials in all neurons that have not spiked yet? Or is this paraphrasing the likely effect of the extremely strong inhibitory synapse (see below)? Also, why should those excitatory neurons "that have" already spiked still be able to generate a spike, this does not make sense to me. Please describe in clear terms!*

This sentence was somewhat misleading. We have corrected this in the revised manuscript. What we mean is that the inhibition is strong enough to reliably suppress firing of excitatory neurons within a time interval of a few milliseconds following the inhibitory spike.

[R2:15] *The very strong inhibitory synaptic current of about -13 nA (Table 2) is problematic because it seems biologically unrealistic. The fact that the authors use a current-based rather than a conductance-based neuron model now becomes relevant. At this point the current based model does no longer approximate the biophysics of a biological neuron where the reversal potential should prevent such large outward charge fluxes and the expected extreme hyperpolarization. This means that the injection of a current profile with -13nA amplitude, resulting in a gigantic charge transfer, is to be considered as a completely artificial intervention. The deliberate effect is probably the complete shutdown of excitatory spiking for a time period longer than the experiment/trial. It would thus be great and transparent to provide (e.g. in supplements) insight in the single neuron propagation/physiology and show in a figure both, the integrated current and membrane potential for e.g. two excitatory and the single inhibitory neuron as a function of time during at least one single learning sequence and a single replay of that sequence. This would allow additional bottom-up insight into the model function that is currently not provided by the more abstract presentation of the few replay spike trains and the derived measures of latencies and relative replay frequencies. The fact that there is only a single inhibitory neuron that is to my interpretation connected to all excitatory neurons ("Excitatory neurons are recurrently connected to the single inhibitory neuron") means a complete suppression of any excitatory output. How could the inhibitory neuron then "meditate competition" (l.198, caption Fig. 3 etc.)? Is this competition only devised during replay (due to 6 times lower synaptic weights?)? This needs to be introduced and explained transparently in the Results and Methods section. Could the authors think of a biologically realistic improvement of their model of inhibition e.g. by introducing a pool of inhibitory neurons, each with a realistic inhibitory effect on the E neurons?*

The purpose of the inhibitory feedback is a) to prevent neurons that are not in the predictive state (i.e., those that have not generated a dAP) to generate a spike in response to the external input (competition between neurons within a given subpopulation), and b) to ensure that, in the presence of ambiguous cues, only one of the competing sequences is replayed (competition between subpopulations). However, this inhibition doesn't have to last very long, only few milliseconds. A single inhibitory neuron (or a population thereof) connected to all excitatory neurons can mediate competition because of the differences in response latencies a) for predictive and non-predictive neurons, and b) for subpopulations that are activated by identical presynaptic subpopulations but with different weights (see Fig.4 in the manuscript). Subpopulations that fire first win. As the inhibition mediates these two different types of competition (a) and b)), it is essential to have it both during learning and during replay.

During learning and during the prediction mode, the external inputs trigger a fast and strong depolarisation of both predictive and non-predictive neurons. Without the inhibition, both types of neurons would fire. Predictive neurons fire somewhat earlier due to the additional depolarization generated by the dAP, as described in the manuscript. In the intact network, this advanced firing triggers a fast and strong inhibition, which prevents the firing of the non-predictive neurons. At the point in time where the inhibitory input arrives at these non-predictive neurons, these neurons are already in a strongly depolarized state (on the way to the spike threshold), far away from the inhibitory reversal potential (which is slightly below the resting potential). Hence, even with conductance-based inhibitory synapses, the inhibition could have a strong resetting effect on these neurons, because the driving force (voltage distance from the reversal potential) is high. The repolarization of these neurons caused by the inhibitory inputs would be limited by the inhibitory reversal potential, but the spike generation would most likely be prevented. A neuron model with conductance based inhibitory synaptic inputs would be more realistic, but we do not expect any qualitative differences to our current-based synapse model with respect to the functioning of the WTA circuit. The risk that the non-predictive neurons still cross the threshold after the inhibition-induced reset could further be reduced by increasing the decay time constant of inhibitory currents such that inhibition has a more long lasting effect. The implementation of the inhibition in our model is simplistic not only with respect to its current-based synapses, but also because we employ only a single inhibitory neuron with very strong and fast outgoing connections. In future versions of the model, one could replace the inhibitory neuron with a recurrently connected network of inhibitory neurons, thereby permitting more realistic inhibitory weights, and simultaneously speeding up the interaction between inhibitory and excitatory cells by virtue of the fast-tracking property of such networks (see Bouhadjar et al., 2022, for a discussion of this scenario). In this scenario, the inhibitory feedback would be fast in the onset but more long lasting because the neurons in the inhibitory network do not fire in perfect synchrony or maintain firing for some time. Such a dispersion in inhibitory spikes would also make the effect of inhibition more long lasting and thereby help suppress non-predictive neurons from responding to the external input.

We have shown voltage traces of predictive (blue) and non-predictive neurons (yellow) in Fig. S6 G–I in (Bouhadjar et al., 2022), see `https://doi.org/10.1371/journal.pcbi.1010233.s007`. Non-predictive neurons (yellow traces) exhibit a strong hyperpolarization in response to the inhibition. In a model with conductance based inhibitory synapses, this hyperpolarization would be bounded by the inhibitory reversal potential.

In the revised manuscript, we have added a new paragraph at the end of the Discussion to discuss the simplifications and possible extensions related to inhibition. In addition, we have added a sentence in the 1st section of Results to emphasize the dual role of inhibition.

[R2:16] 5. Neuron dynamics *I understand that all neurons are point neurons and the voltage dynamics is governed by eqn 6 and all input currents simply add up (eqn 7). Therefore, the term "dendritic current"* I_{ED} *really is only an interpretation and not reflected in any distal input with length constants or compartments of a dendrite with geometry. The reasoning behind the properties of* I_ED *needs to be outlined and the authors should make more explicit that this is why they interpret these inputs as the somatic effect of distal dendritic inputs. Currently, the authors make no claims and do not provide references to why the properties of* I_ED *represent a somatic effect of distal excitatory dendritic inputs. Specifically, why is the PSC alpha-shaped, should this reflect a dendritic filtering and if so, should it not be a beta-shaped current with a second time constant? More important, why is the dendritic AP represented by a constant*

plateau current (that is seemingly not filtered or damped during its travel to the soma)? Is the biological motivation the current effect of a dendritic Ca spike or a dendritic Na spike or something else? Please clarify in the text. The fact that the threshold is applied to the sum of all dendritic inputs means that the dAP is not dendritic branch specific, a yet unexplored input dimension of the model.

The reviewer is right: we model neurons as leaky integrate-and fire point neurons, but with a nonlinear integration of local excitatory inputs to excitatory neurons. We label (interpret) these inputs as "dendritic", i.e., we assume in this model that excitatory neurons target other excitatory neurons on distal dendritic branches, such that they can elicit NMDA spikes (dAPs). Effects of the neuron morphology, such as the length constants of dendritic compartments, are described indirectly in an abstract, phenomenological manner. Similar models have ben used in previous theoretical studies such as (Jahnke et al., 2012; Breuer et al., 2014).

Using alpha-function shaped PSCs for the dendritic currents is certainly reasonable due to dendritic filtering. Here, however, there is a more fundamental reason for this choice: we need alpha-function shaped PSCs to make sure that the response latencies during replay depend on the synaptic weights (see Fig.4A and B). The amplitude of the plateau current I_{dAP}, which is triggered by the dAP-threshold crossing, is constant and does not dependent on the synaptic weights. For exponential PSCs with an instantaneous increment, the arrival of synchronous presynaptic spikes leads to an instantaneous dAP-threshold crossing in the synaptic current, and hence, and instantaneous clamping of the current to the plateau current I_{dAP}. Hence, neither the onset time nor the amplitude of this plateau current depends on the synaptic weights (provided the synaptic weights are strong enough to trigger a dAP). Therefore, also the membrane potential in response to this dendritic current does not carry any information about the synaptic weights. In consequence, the response latencies during replay would become independent of the weights, and hence, of the training frequencies. With alpha-function shaped dendritic PSCs, in contrast, the time to the dAP-threshold becomes weight dependent due to the finite rise time.

The assumption that the dAP plateau current is constant for the entire duration of the dAP and not subject to dendritic filtering is a simplification. We are not aiming at a realistic description of the dAP-related current itself, but only its effect on the somatic membrane potential. Similarly, we restrict this study to a single dendritic branch per excitatory neuron. Equipping neurons with multiple active dendritic branches would lead to an increase in the network capacity, as each branch constitutes an independent pattern detector (Ahmad & Hawkins, 2016; Hawkins & Ahmad, 2016). We leave this extension of the model to future studies.

In the revised manuscript, we have extended the description of the "Neuron and synapse model" in the Methods to address the reviewer's comments.

[R2:17] 6 Latency analysis unclear *I was a little confused with the latency analysis. The definition in line 273ff seems to be restricted to a single subscript index "s". Do the latencies in Fig. 4 show latencies to one particular stimulus in the sequence or the average latency? In l.273 "(time of first spike after the cue)" – this is unclear: The absolute numbers of latency in the order of 50ms together with the spike times in Fig. 3B-D indicate that the latency is computed from the spike times relative to the *previous* cue/stimulus and not the present? Please clarify. Related to this: in l.237ff subscript "s" is not defined. Does it denote the number or the type of stimulus in a sequence? Ambiguity with sequences denoted as s1, s2, . . . (l. 177) is possible. The symbol „t" is used for timing of stimuli/cues (l.121) and for spike time and for the average spike latency (l.273). What is the difference between subscript and superscript indices i,j in lines*

120-124 and lines 127 and 247f, this is confusing. Is superscript type and subscript number of stimulus within a sequence?

We agree that the notation in these definitions was confusing. In the revised manuscript, we have simplified this and refer to t_i (without the subscript "s") as the time of the first spike of neuron i after the cue. However, we keep the subscript "s" in v_s and c_s to make clear that these statistics refer to the (co-)variability in the (first) "spike" times, rather than in the input currents. We added a note in the text below equation (2) explaining this.

Note that, in the revised manuscript, we have moved the definition of the response latency (equation (1)) to the previous paragraph where this quantity is already introduced. Further, we refer to this definition in the caption of Fig.4. We hope this makes it clearer.

The latencies in Fig.4B,C,D represent the mean (symbols) and standard deviation (error bars) of the population-averaged latencies $t_{\mathrm{B/C}}$ across 150 trials (stimulus repetitions), subsequently averaged across 5 different network realizations.

All latencies are measured relative to the cue onset in each trial, which is the presentation of (ambiguous) character "A" in the examples. Apart from the presentation of this first character (the cue), there are no further stimuli. In the examples, subpopulations "B" and "C" correspond to the 3rd elements in the two competing sequences. After stimulating "A", the activity propagates to "F" and from there to "B" and "C". Hence, there are two synapses between "A" and "B/C", resulting in total latencies $t^{\mathrm{B/C}} \sim 50\,\mathrm{ms}$ (see Fig.3B–D).

[R2:18] *Minor:*
The Ordering of "Materials & Methods" (1st) and "Results" (2nd) sections does not adhere to the guidelines of PLoS CB. At the end of the Introduction the authors point out, that the reader may proceed to the Results section. This is not necessary if the order is reversed and this might refer to an earlier submission elsewhere.

The reviewer is right. We corrected the order and applied the necessary modifications.

[R2:19]

- *Caption Fig. 1: "two sequences A,F,C,E (brown) and A,F,B,D (blue)" – colors are exchanged.*
- *l.157: "(9-10)" − > "Eq. (9-10)"*
- *l.157: "The weights J_{IE} of excitatory synapses on inhibitory neurons..:" – should be singular "inhibitory neuron".*

We thank the reviewer for pointing out these issues. We corrected these now.

[R2:20] *Is "cue" and "stimulus" used interchangeable or are they defined differently?*

In this study, we consider two different types of external stimuli: during training, the network is stimulated for each sequence element occurring in the set of sequences to be learned. During replay, only the first (ambiguous) sequence element is presented, constituting a "cue". Throughout this manuscript, we use the term "cue" or "cue stimulus" when referring to the latter type of stimulus. Both types of stimuli are presented to the network via external spike sources. In the revised manuscript, we improved the description of these stimuli in the Methods, paragraphs

"External inputs during learning/replay".

[R2:21] *Towards the end of the discussion the authors argue that "The total input to a neuron resulting from large ensembles of synapses, however, is likely to be subject to noise averaging, unless the variability of synaptic responses is correlated across synapses." To this end and in line with the authors' authors argument on averaging of independent (synaptic) noise the following reference provides a combined experimental-theoretical analysis of the effect of synaptic amplitude variability on postsynaptic input summation:*

- *Nawrot, M. P., Schnepel, P., Aertsen, A., & Boucsein, C. (2009). Precisely timed signal transmission in neocortical networks with reliable intermediate-range projections. Frontiers in Neural Circuits, 1.*

We thank the reviewer for pointing this out. In the revised manuscript, we now refer to the work of Nawrot et al. (2009) in the 2nd last paragraph of the Discussion, in support of our argument that "synaptic stochasticity has only a marginal effect on the variability of postsynaptic responses."

References

Bouhadjar, Y., Wouters, D. J., Diesmann, M., & Tetzlaff, T. (2022). Sequence learning, prediction, and replay in networks of spiking neurons. *PLOS Computational Biology 18*(6), e1010233.

Hawkins, J., & Ahmad, S. (2016). Why neurons have thousands of synapses, a theory of sequence memory in neocortex. *Frontiers in Neural Circuits 10*, 23.

Ahmad, S., & Hawkins, J. (2016). How do neurons operate on sparse distributed representations? a mathematical theory of sparsity, neurons and active dendrites. *arXiv*, 1601.00720.

Jahnke, S., Timme, M., & Memmesheimer, R.-M. (2012). Guiding synchrony through random networks. *Physical Review X 2*(4), 041016.

Breuer, D., Timme, M., & Memmesheimer, R.-M. (2014). Statistical physics of neural systems with nonadditive dendritic coupling. *Physical Review X 4*(1), 011053.
